# *trans*-Tetra­aqua­bis{(*E*)-2-cyano-1-[(ethoxy­carbon­yl)methyl­sulfan­yl]-2-(1-naphthyl­amino­carbon­yl)ethene-1-thiol­ato}calcium(II) diethyl ether disolvate

**DOI:** 10.1107/S1600536810013024

**Published:** 2010-04-21

**Authors:** Galal H. Elgemeie, Nahed M. Fathy, Sayed Shaarawi, Peter G. Jones

**Affiliations:** a Chemistry Department, Faculty of Science, Helwan University, Ain-Helwan, Helwan, Egypt; b National Research Centre, Dokki, Cairo, Egypt; c Institut für Anorganische und Analytische Chemie, Technische Universität Braunschweig, Postfach 3329, 38023 Braunschweig, Germany

## Abstract

In the title compound, [Ca(C_18_H_15_N_2_O_3_S_2_)(H_2_O)_4_]·2C_4_H_10_O, the Ca atom, which lies on an inversion centre, is coordinated octa­hedrally by four water mol­ecules and two anions of the ketene dithio­acetal, the donor atoms of which are the amidic carbonyl O atoms. The central backbone of the ligands (excluding the naphthalene and oxoethyl groups) is essentially planar (r.m.s. deviation 0.035 Å). Intra­molecular hydrogen bonds are observed from the NH group to the formally ‘thiol­ate’ S atom and from one coordinated water to the nitrile group and to the ether O atom. Inter­molecular hydrogen bonds from the second independent water mol­ecule to the thiol­ate S atom and the side-chain oxo group connect the mol­ecules in chains parallel to the *a* axis.

## Related literature

For our studies exploring the synthetic potential of ketene dithioacetals for synthesizing new classes of novel antimetabolic agents, see: Elgemeie & Sood (2006 ▶); Elgemeie *et al.* (2008 ▶, 2009 ▶). For our reports of successful approaches for the synthesis of mercaptopurine and pyrimidine analogues by the reaction of ketene dithioacetals with active methylene functions, see: Elgemeie (2003 ▶); Elgemeie *et al.* (2004 ▶, 2007 ▶).
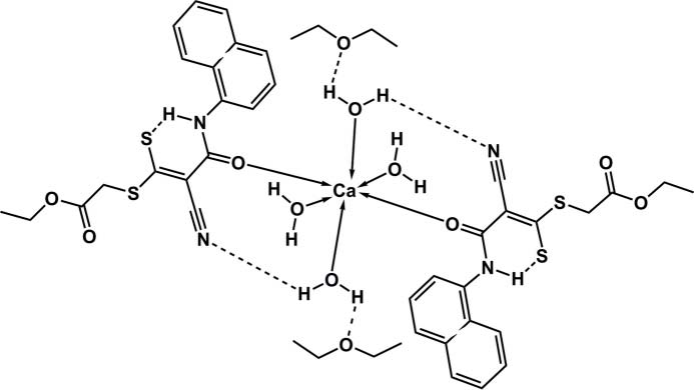


## Experimental

### 

#### Crystal data

[Ca(C_18_H_15_N_2_O_3_S_2_)(H_2_O)_4_]·2C_4_H_10_O
*M* *_r_* = 1003.26
Triclinic, 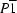

*a* = 7.8665 (3) Å
*b* = 12.4361 (5) Å
*c* = 13.8045 (6) Å
α = 103.692 (4)°
β = 99.963 (4)°
γ = 101.609 (3)°
*V* = 1250.24 (9) Å^3^
*Z* = 1
Mo *K*α radiation
μ = 0.35 mm^−1^
*T* = 100 K
0.4 × 0.3 × 0.1 mm

#### Data collection

Oxford Diffraction Xcalibur Eos diffractometer
Absorption correction: multi-scan (*CrysAlis PRO*; Oxford Diffraction, 2009 ▶) *T*_min_ = 0.970, *T*_max_ = 1.000
26853 measured reflections
6443 independent reflections
4941 reflections with *I* > 2σ(*I*)
*R*_int_ = 0.031

#### Refinement

*R*[*F* ^2^ > 2σ(*F* ^2^)] = 0.029
*wR*(*F* ^2^) = 0.070
*S* = 0.96
6443 reflections
326 parameters
18 restraints
H atoms treated by a mixture of independent and constrained refinement
Δρ_max_ = 0.30 e Å^−3^
Δρ_min_ = −0.28 e Å^−3^

### 

Data collection: *CrysAlis PRO* (Oxford Diffraction, 2009 ▶); cell refinement: *CrysAlis PRO*; data reduction: *CrysAlis PRO*; program(s) used to solve structure: *SHELXS97* (Sheldrick, 2008 ▶); program(s) used to refine structure: *SHELXL97* (Sheldrick, 2008 ▶); molecular graphics: *XP* (Siemens, 1994 ▶); software used to prepare material for publication: *SHELXL97*.

## Supplementary Material

Crystal structure: contains datablocks I, global. DOI: 10.1107/S1600536810013024/bt5223sup1.cif

Structure factors: contains datablocks I. DOI: 10.1107/S1600536810013024/bt5223Isup2.hkl

Additional supplementary materials:  crystallographic information; 3D view; checkCIF report

## Figures and Tables

**Table 1:** Selected bond lengths (Å)

Ca—O1	2.2696 (9)
Ca—O2*W*	2.3265 (10)
Ca—O1*W*	2.3434 (10)
S1—C13	1.7686 (12)
S1—C14	1.7891 (12)
S2—C13	1.7037 (12)
O1—C11	1.2494 (14)
N1—C11	1.3448 (15)
N1—C1	1.4184 (15)
N2—C18	1.1518 (16)
C11—C12	1.4684 (16)
C12—C13	1.3996 (17)
C12—C18	1.4325 (17)

**Table 2:** Hydrogen-bond geometry (Å, °)

*D*—H⋯*A*	*D*—H	H⋯*A*	*D*⋯*A*	*D*—H⋯*A*
N1—H01⋯S2	0.855 (16)	2.279 (16)	3.0274 (11)	146.2 (14)
O1*W*—H1*W*⋯O93	0.82 (1)	1.94 (1)	2.7531 (14)	171 (2)
O1*W*—H2*W*⋯N2^i^	0.82 (1)	2.22 (1)	2.9902 (15)	157 (2)
O2*W*—H3*W*⋯O3^ii^	0.82 (1)	2.03 (1)	2.7797 (13)	151 (2)
O2*W*—H4*W*⋯S2^ii^	0.81 (1)	2.51 (1)	3.2666 (11)	157 (2)

Symmetry codes: (i) 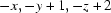; (ii) 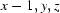.

**Fig. 1.**
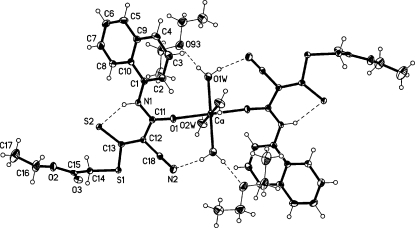
View of the title compound showing ellipsoids at the 50% probability level. Intramolecular hydrogen bonds are indicated by thin dashed lines.

**Fig. 2.**
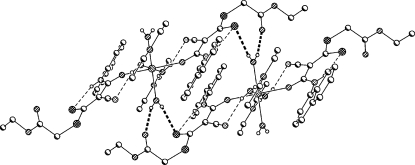
Packing diagram of the title compound showing two molecules connected by intermolecular hydrogen bonds (thick dashed lines).

## supplementary crystallographic information

### Comment

During the course of our studies directed toward exploring the synthetic potential of ketene dithioacetals for synthesizing new classes of novel antimetabolic agents (Elgemeie & Sood, 2006; Elgemeie *et al.*, 2008, 2009), we have recently reported various successful approaches for synthesis of mercaptopurine and pyrimidine analogues by the reaction of ketene dithioacetals with active methylene functions (Elgemeie, 2003; Elgemeie *et al.*, 2004, 2007). In conjunction of this work, we report here a novel calcium salt (I) of a ketene dithioacetal. The structure of (I) was established on the basis of its elemental analysis and spectral data (see Experimental). In order to establish unambiguously the structure of the product, the crystal structure was determined. The X-ray analysis confirms the exclusive presence of the form (I) in the solid state (Figure 1).

The structure consists of a calcium ion on an inversion centre, coordinated octahedrally by four water molecules and two anions of the ketene dithioacetal; the latter coordinate via the amidic carbonyl oxygen O1, with Ca—O1 2.2696 (9) Å. The asymmetric unit also contains one molecule of diethyl ether.

Within the ligand, there is presumably extensive delocalization of the negative charge from its formal position at the "thiolate" sulfur S2 through the ligand backbone (see dimensions in Table 1), in which the ten atoms C1, C11–14, C18, N1, O1, S1 and S2 are approximately coplanar (r.m.s. deviation 0.035 Å). The backbone angles C11—C12—C13 and C12—C13—S2 are noticeably wide at 126.43 (9) and 129.48 (11)° respectively. The Ca atom lies 0.862 (1) Å out of the backbone plane,and the naphthalene plane subtends an interplanar angle to this plane of 29.03 (2)°.

Intramolecular hydrogen bonds (Table 2) are observed from the NH function to the formally thiolate sulfur and from a hydrogen of the coordinated water O1W to the nitrile N. Additionally, the ether molecules are connected by a hydrogen bond from the same water to the ether oxygen. Intramolecular H bonds from the second water to the thiolate sulfur and the 2-oxo function connect the molecules to form chains parallel to the *a* axis (Fig. 2).

### Experimental

The IR spectrum revealed the presence of cyano group at 2190 cm^-1^. The ^1^H NMR spectrum revealed signals at δ 1.06–1.24 ppm (t, CH_3_), 2.51 (s, SCH_2_), 4.08–4.15 (qua, ester CH_2_) 7.43–8.37 (m, aromatic protons) and 8.40 (br s, NH).

### Refinement

NH and OH hydrogens were identified in difference syntheses. The NH hydrogen was refined freely, water H freely but with distance restraints (SADI) to O—H and H···H. Methyl protons were identified in difference syntheses, idealised and allowed to refine as rigid groups (C—H 0.98 Å, H—C—H 109.5°) allowed to rotate but not tip. Other H were included starting from calculated, idealised positions using a riding model with C—H 0.99 Å for methylene groups and 0.95 Å for *sp*^2^ carbons. The ethyl group C16/17 is disordered over two positions with occupancies 0.738, 0.262 (4). Similarity restraints to this group were used to improve stability of refinement.

### Crystal data

**Table:**

[Ca(C_18_H_15_N_2_O_3_S_2_)(H_2_O)_4_]·2C_4_H_10_O	*F*(000) = 530
*M**_r_* = 1003.26	*D*_x_ = 1.333 Mg m^−^^3^
Triclinic, *P*1	Melting point = 479–481 K
*a* = 7.8665 (3) Å	Mo *K*α radiation, λ = 0.71073 Å
*b* = 12.4361 (5) Å	Cell parameters from 11577 reflections
*c* = 13.8045 (6) Å	θ = 2.6–30.8°
α = 103.692 (4)°	µ = 0.35 mm^−^^1^
β = 99.963 (4)°	*T* = 100 K
γ = 101.609 (3)°	Irregular tablet, pale yellow
*V* = 1250.24 (9) Å^3^	0.4 × 0.3 × 0.1 mm
*Z* = 1	

### Data collection

**Table:**

Oxford Diffraction Xcalibur Eos diffractometer	6443 independent reflections
Radiation source: Enhance (Mo) X-ray Source	4941 reflections with *I* > 2σ(*I*)
graphite	*R*_int_ = 0.031
Detector resolution: 16.1419 pixels mm^-1^	θ_max_ = 28.7°, θ_min_ = 2.7°
ω–scan	*h* = −10→10
Absorption correction: multi-scan (*CrysAlis PRO*; Oxford Diffraction, 2009)	*k* = −16→16
*T*_min_ = 0.970, *T*_max_ = 1.000	*l* = −18→18
26853 measured reflections	

### Refinement

**Table:**

Refinement on *F*^2^	Primary atom site location: structure-invariant direct methods
Least-squares matrix: full	Secondary atom site location: difference Fourier map
*R*[*F*^2^ > 2σ(*F*^2^)] = 0.029	Hydrogen site location: inferred from neighbouring sites
*wR*(*F*^2^) = 0.070	H atoms treated by a mixture of independent and constrained refinement
*S* = 0.96	*w* = 1/[σ^2^(*F*_o_^2^) + (0.0363*P*)^2^] where *P* = (*F*_o_^2^ + 2*F*_c_^2^)/3
6443 reflections	(Δ/σ)_max_ = 0.001
326 parameters	Δρ_max_ = 0.30 e Å^−^^3^
18 restraints	Δρ_min_ = −0.28 e Å^−^^3^

### Special details

**Table:**

Geometry. All esds (except the esd in the dihedral angle between two l.s. planes) are estimated using the full covariance matrix. The cell esds are taken into account individually in the estimation of esds in distances, angles and torsion angles; correlations between esds in cell parameters are only used when they are defined by crystal symmetry. An approximate (isotropic) treatment of cell esds is used for estimating esds involving l.s. planes.Least-squares planes (x,y,z in crystal coordinates) and deviations from them (* indicates atom used to define plane)5.7234 (0.0013) x - 5.6563 (0.0020) y + 7.4193 (0.0030) z = 5.4532 (0.0027)* 0.0098 (0.0008) C1 * 0.0042 (0.0011) C11 * 0.0033 (0.0011) C12 * -0.0107 (0.0010) C13 * 0.0215 (0.0007) C14 * 0.0075 (0.0008) C18 * 0.0664 (0.0009) N1 * -0.0508 (0.0008) O1 * 0.0150 (0.0006) S1 * -0.0662 (0.0006) S2 -0.8620 (0.0010) CaRms deviation of fitted atoms = 0.03517.5293 (0.0008) x - 5.2398 (0.0041) y + 0.8431 (0.0033) z = 1.0859 (0.0024)Angle to previous plane (with approximate esd) = 29.03 ( 0.02 )* -0.0015 (0.0010) C1 * 0.0157 (0.0010) C2 * 0.0006 (0.0010) C3 * -0.0152 (0.0011) C4 * 0.0063 (0.0011) C5 * 0.0109 (0.0011) C6 * -0.0015 (0.0011) C7 * -0.0092 (0.0010) C8 * -0.0036 (0.0011) C9 * -0.0025 (0.0011) C10Rms deviation of fitted atoms = 0.0086
Refinement. Refinement of *F*^2^ against ALL reflections. The weighted *R*-factor wR and goodness of fit *S* are based on *F*^2^, conventional *R*-factors *R* are based on *F*, with *F* set to zero for negative *F*^2^. The threshold expression of *F*^2^ > σ(*F*^2^) is used only for calculating *R*-factors(gt) etc. and is not relevant to the choice of reflections for refinement. *R*-factors based on *F*^2^ are statistically about twice as large as those based on *F*, and *R*- factors based on ALL data will be even larger.

### Fractional atomic coordinates and isotropic or equivalent isotropic displacement parameters (Å^2^)

**Table:**

	*x*	*y*	*z*	*U*_iso_*/*U*_eq_	Occ. (<1)
Ca	0.0000	0.5000	1.0000	0.01453 (8)	
S1	0.64002 (4)	0.90145 (3)	0.93054 (2)	0.01677 (8)	
S2	0.63380 (4)	0.69288 (3)	0.76538 (2)	0.01590 (8)	
O1	0.25364 (11)	0.53927 (7)	0.94360 (7)	0.0198 (2)	
O3	1.01513 (11)	0.87570 (7)	0.93853 (7)	0.0201 (2)	
N1	0.39935 (14)	0.49923 (9)	0.81647 (8)	0.0156 (2)	
H01	0.471 (2)	0.5286 (13)	0.7844 (11)	0.032 (4)*	
N2	0.36142 (15)	0.81149 (9)	1.07579 (8)	0.0225 (2)	
C1	0.32121 (15)	0.37980 (10)	0.77809 (9)	0.0152 (3)	
C2	0.27106 (16)	0.31450 (10)	0.84051 (10)	0.0173 (3)	
H2	0.2901	0.3491	0.9120	0.021*	
C3	0.19158 (17)	0.19658 (11)	0.79947 (10)	0.0211 (3)	
H3	0.1560	0.1523	0.8433	0.025*	
C4	0.16515 (17)	0.14516 (11)	0.69724 (10)	0.0210 (3)	
H4	0.1102	0.0656	0.6706	0.025*	
C5	0.19573 (17)	0.15718 (11)	0.52430 (10)	0.0236 (3)	
H5	0.1437	0.0773	0.4969	0.028*	
C6	0.24685 (18)	0.21949 (12)	0.46050 (10)	0.0256 (3)	
H6	0.2309	0.1830	0.3897	0.031*	
C7	0.32322 (17)	0.33790 (12)	0.49968 (10)	0.0240 (3)	
H7	0.3580	0.3813	0.4550	0.029*	
C8	0.34779 (17)	0.39113 (11)	0.60195 (9)	0.0193 (3)	
H8	0.3994	0.4712	0.6272	0.023*	
C9	0.21843 (16)	0.20880 (10)	0.63064 (10)	0.0178 (3)	
C10	0.29770 (15)	0.32891 (10)	0.67054 (9)	0.0153 (3)	
C11	0.36103 (16)	0.57231 (10)	0.89338 (9)	0.0143 (2)	
C12	0.44847 (15)	0.69477 (10)	0.91915 (9)	0.0139 (2)	
C13	0.56499 (15)	0.75240 (10)	0.87132 (9)	0.0137 (2)	
C14	0.78632 (16)	0.94911 (10)	0.85488 (9)	0.0147 (2)	
H14A	0.7223	0.9213	0.7818	0.018*	
H14B	0.8187	1.0338	0.8744	0.018*	
C15	0.95496 (16)	0.90825 (10)	0.86744 (9)	0.0152 (3)	
O2	1.03244 (11)	0.91600 (8)	0.78997 (7)	0.0246 (2)	
C16	1.1903 (3)	0.8657 (3)	0.7942 (2)	0.0354 (7)	0.738 (4)
H16A	1.2878	0.9141	0.8533	0.042*	0.738 (4)
H16B	1.1571	0.7883	0.8036	0.042*	0.738 (4)
C17	1.2505 (3)	0.8589 (2)	0.69962 (17)	0.0436 (8)	0.738 (4)
H17A	1.3527	0.8250	0.7021	0.065*	0.738 (4)
H17B	1.2859	0.9359	0.6916	0.065*	0.738 (4)
H17C	1.1533	0.8113	0.6414	0.065*	0.738 (4)
C16'	1.2112 (11)	0.9059 (6)	0.7918 (7)	0.029 (2)*	0.262 (4)
H16C	1.2780	0.9128	0.8616	0.034*	0.262 (4)
H16D	1.2780	0.9625	0.7633	0.034*	0.262 (4)
C17'	1.1676 (9)	0.7828 (6)	0.7206 (5)	0.044 (2)*	0.262 (4)
H17D	1.2788	0.7609	0.7139	0.066*	0.262 (4)
H17E	1.0996	0.7794	0.6529	0.066*	0.262 (4)
H17F	1.0967	0.7301	0.7499	0.066*	0.262 (4)
C18	0.40096 (16)	0.75993 (10)	1.00605 (9)	0.0152 (2)	
C91	−0.0616 (2)	0.49289 (14)	0.64737 (12)	0.0380 (4)	
H91A	0.0392	0.4925	0.7000	0.057*	
H91B	−0.1516	0.5204	0.6803	0.057*	
H91C	−0.0198	0.5436	0.6071	0.057*	
C92	−0.1421 (2)	0.37394 (14)	0.57815 (11)	0.0314 (3)	
H92A	−0.2440	0.3737	0.5247	0.038*	
H92B	−0.0523	0.3460	0.5439	0.038*	
O93	−0.20128 (13)	0.30117 (9)	0.63788 (7)	0.0283 (2)	
C94	−0.2807 (2)	0.18547 (13)	0.57820 (11)	0.0332 (4)	
H94A	−0.1931	0.1539	0.5444	0.040*	
H94B	−0.3842	0.1823	0.5244	0.040*	
C95	−0.3405 (2)	0.11591 (14)	0.64723 (12)	0.0375 (4)	
H95A	−0.4274	0.1474	0.6803	0.056*	
H95B	−0.2373	0.1185	0.6997	0.056*	
H95C	−0.3959	0.0364	0.6067	0.056*	
O1W	−0.17859 (14)	0.37346 (9)	0.84531 (8)	0.0318 (3)	
H1W	−0.173 (2)	0.3536 (15)	0.7848 (10)	0.051 (6)*	
H2W	−0.252 (2)	0.3209 (14)	0.8526 (14)	0.064 (7)*	
O2W	−0.07611 (14)	0.64913 (9)	0.94233 (8)	0.0277 (2)	
H3W	−0.023 (2)	0.7169 (11)	0.9595 (12)	0.044 (5)*	
H4W	−0.155 (2)	0.6389 (15)	0.8925 (11)	0.048 (6)*	

### Atomic displacement parameters (Å^2^)

**Table:**

	*U*^11^	*U*^22^	*U*^33^	*U*^12^	*U*^13^	*U*^23^
Ca	0.01563 (18)	0.01361 (17)	0.01734 (18)	0.00374 (13)	0.00750 (14)	0.00726 (13)
S1	0.01826 (16)	0.01288 (15)	0.01747 (16)	0.00078 (12)	0.00818 (12)	0.00085 (12)
S2	0.01890 (16)	0.01387 (15)	0.01454 (15)	0.00153 (12)	0.00815 (12)	0.00241 (11)
O1	0.0199 (5)	0.0174 (4)	0.0240 (5)	0.0023 (4)	0.0127 (4)	0.0061 (4)
O3	0.0200 (5)	0.0161 (4)	0.0215 (5)	0.0033 (4)	−0.0013 (4)	0.0058 (4)
N1	0.0165 (5)	0.0134 (5)	0.0162 (5)	0.0004 (4)	0.0076 (4)	0.0032 (4)
N2	0.0280 (6)	0.0196 (6)	0.0241 (6)	0.0076 (5)	0.0136 (5)	0.0074 (5)
C1	0.0118 (6)	0.0143 (6)	0.0184 (6)	0.0025 (5)	0.0029 (5)	0.0036 (5)
C2	0.0182 (6)	0.0168 (6)	0.0167 (6)	0.0039 (5)	0.0037 (5)	0.0050 (5)
C3	0.0216 (7)	0.0176 (6)	0.0262 (7)	0.0036 (5)	0.0064 (6)	0.0107 (5)
C4	0.0184 (7)	0.0131 (6)	0.0277 (7)	0.0004 (5)	0.0020 (5)	0.0038 (5)
C5	0.0192 (7)	0.0198 (7)	0.0242 (7)	0.0025 (5)	0.0018 (6)	−0.0037 (5)
C6	0.0244 (7)	0.0304 (8)	0.0165 (7)	0.0049 (6)	0.0045 (6)	−0.0017 (6)
C7	0.0243 (7)	0.0288 (7)	0.0189 (7)	0.0048 (6)	0.0079 (6)	0.0063 (6)
C8	0.0201 (7)	0.0181 (6)	0.0179 (6)	0.0017 (5)	0.0056 (5)	0.0032 (5)
C9	0.0133 (6)	0.0166 (6)	0.0211 (7)	0.0035 (5)	0.0022 (5)	0.0022 (5)
C10	0.0111 (6)	0.0168 (6)	0.0166 (6)	0.0032 (5)	0.0024 (5)	0.0031 (5)
C11	0.0129 (6)	0.0166 (6)	0.0142 (6)	0.0043 (5)	0.0035 (5)	0.0050 (5)
C12	0.0128 (6)	0.0154 (6)	0.0135 (6)	0.0036 (5)	0.0041 (5)	0.0030 (5)
C13	0.0121 (6)	0.0143 (6)	0.0135 (6)	0.0034 (5)	0.0013 (5)	0.0029 (5)
C14	0.0165 (6)	0.0130 (6)	0.0155 (6)	0.0029 (5)	0.0051 (5)	0.0050 (5)
C15	0.0152 (6)	0.0091 (5)	0.0174 (6)	−0.0010 (5)	0.0025 (5)	0.0008 (5)
O2	0.0161 (5)	0.0383 (6)	0.0213 (5)	0.0076 (4)	0.0079 (4)	0.0089 (4)
C16	0.0198 (12)	0.055 (2)	0.0368 (14)	0.0198 (14)	0.0121 (10)	0.0105 (15)
C17	0.0438 (15)	0.0669 (19)	0.0368 (13)	0.0383 (13)	0.0182 (11)	0.0195 (12)
C18	0.0151 (6)	0.0138 (6)	0.0184 (6)	0.0032 (5)	0.0056 (5)	0.0072 (5)
C91	0.0465 (10)	0.0441 (10)	0.0394 (9)	0.0246 (8)	0.0220 (8)	0.0223 (8)
C92	0.0300 (8)	0.0499 (10)	0.0230 (7)	0.0173 (7)	0.0103 (6)	0.0173 (7)
O93	0.0310 (6)	0.0366 (6)	0.0186 (5)	0.0095 (4)	0.0078 (4)	0.0078 (4)
C94	0.0259 (8)	0.0439 (9)	0.0240 (8)	0.0094 (7)	0.0017 (6)	0.0011 (7)
C95	0.0320 (9)	0.0389 (9)	0.0366 (9)	0.0044 (7)	0.0073 (7)	0.0056 (7)
O1W	0.0370 (6)	0.0330 (6)	0.0181 (5)	−0.0079 (5)	0.0103 (5)	0.0050 (5)
O2W	0.0291 (6)	0.0183 (5)	0.0353 (6)	0.0044 (4)	0.0007 (5)	0.0133 (5)

### Geometric parameters (Å, °)

**Table:**

Ca—O1	2.2696 (9)	N1—H01	0.855 (16)
Ca—O2W	2.3265 (10)	C2—H2	0.9500
Ca—O1W	2.3434 (10)	C3—H3	0.9500
S1—C13	1.7686 (12)	C4—H4	0.9500
S1—C14	1.7891 (12)	C5—H5	0.9500
S2—C13	1.7037 (12)	C6—H6	0.9500
O1—C11	1.2494 (14)	C7—H7	0.9500
O3—C15	1.2064 (15)	C8—H8	0.9500
N1—C11	1.3448 (15)	C14—H14A	0.9900
N1—C1	1.4184 (15)	C14—H14B	0.9900
N2—C18	1.1518 (16)	C16—H16A	0.9900
C1—C2	1.3702 (17)	C16—H16B	0.9900
C1—C10	1.4325 (17)	C17—H17A	0.9800
C2—C3	1.4063 (17)	C17—H17B	0.9800
C3—C4	1.3643 (18)	C17—H17C	0.9800
C4—C9	1.4113 (18)	C16'—H16C	0.9900
C5—C6	1.3631 (19)	C16'—H16D	0.9900
C5—C9	1.4199 (18)	C17'—H17D	0.9800
C6—C7	1.4059 (19)	C17'—H17E	0.9800
C7—C8	1.3713 (17)	C17'—H17F	0.9800
C8—C10	1.4156 (17)	C91—H91A	0.9800
C9—C10	1.4284 (16)	C91—H91B	0.9800
C11—C12	1.4684 (16)	C91—H91C	0.9800
C12—C13	1.3996 (17)	C92—H92A	0.9900
C12—C18	1.4325 (17)	C92—H92B	0.9900
C14—C15	1.5105 (17)	C94—H94A	0.9900
C15—O2	1.3323 (15)	C94—H94B	0.9900
O2—C16'	1.433 (9)	C95—H95A	0.9800
O2—C16	1.497 (3)	C95—H95B	0.9800
C16—C17	1.455 (3)	C95—H95C	0.9800
C16'—C17'	1.545 (8)	O1W—H1W	0.824 (13)
C91—C92	1.499 (2)	O1W—H2W	0.820 (13)
C92—O93	1.4255 (17)	O2W—H3W	0.819 (13)
O93—C94	1.4277 (17)	O2W—H4W	0.810 (13)
C94—C95	1.503 (2)		
			
O1^i^—Ca—O1	180.0	C9—C5—H5	119.2
O1—Ca—O2W^i^	92.96 (4)	C5—C6—H6	120.1
O1^i^—Ca—O2W	92.96 (4)	C7—C6—H6	120.1
O1—Ca—O2W	87.04 (4)	C8—C7—H7	119.8
O2W^i^—Ca—O2W	180.0	C6—C7—H7	119.8
O1—Ca—O1W^i^	83.15 (3)	C7—C8—H8	119.4
O2W—Ca—O1W^i^	91.93 (4)	C10—C8—H8	119.4
O1^i^—Ca—O1W	83.15 (4)	C15—C14—H14A	109.0
O1—Ca—O1W	96.85 (3)	S1—C14—H14A	109.0
O2W^i^—Ca—O1W	91.93 (4)	C15—C14—H14B	109.0
O2W—Ca—O1W	88.07 (4)	S1—C14—H14B	109.0
O1W^i^—Ca—O1W	180.0	H14A—C14—H14B	107.8
C13—S1—C14	103.00 (6)	C17—C16—H16A	109.8
C11—O1—Ca	161.36 (9)	O2—C16—H16A	109.8
C11—N1—C1	126.41 (11)	C17—C16—H16B	109.8
C2—C1—N1	122.01 (11)	O2—C16—H16B	109.8
C2—C1—C10	120.73 (11)	H16A—C16—H16B	108.2
N1—C1—C10	117.26 (11)	C16—C17—H17A	109.5
C1—C2—C3	120.42 (12)	C16—C17—H17B	109.5
C4—C3—C2	120.61 (12)	H17A—C17—H17B	109.5
C3—C4—C9	120.80 (12)	C16—C17—H17C	109.5
C6—C5—C9	121.59 (12)	H17A—C17—H17C	109.5
C5—C6—C7	119.90 (12)	H17B—C17—H17C	109.5
C8—C7—C6	120.40 (13)	O2—C16'—H16C	112.1
C7—C8—C10	121.21 (12)	C17'—C16'—H16C	112.1
C4—C9—C5	122.03 (12)	O2—C16'—H16D	112.1
C4—C9—C10	119.51 (11)	C17'—C16'—H16D	112.1
C5—C9—C10	118.46 (12)	H16C—C16'—H16D	109.7
C8—C10—C9	118.44 (11)	C16'—C17'—H17D	109.5
C8—C10—C1	123.63 (11)	C16'—C17'—H17E	109.5
C9—C10—C1	117.93 (11)	H17D—C17'—H17E	109.5
O1—C11—N1	122.08 (11)	C16'—C17'—H17F	109.5
O1—C11—C12	119.03 (11)	H17D—C17'—H17F	109.5
N1—C11—C12	118.89 (11)	H17E—C17'—H17F	109.5
C13—C12—C18	118.58 (11)	C92—C91—H91A	109.5
C13—C12—C11	129.48 (11)	C92—C91—H91B	109.5
C18—C12—C11	111.94 (10)	H91A—C91—H91B	109.5
C12—C13—S2	126.43 (9)	C92—C91—H91C	109.5
C12—C13—S1	113.61 (9)	H91A—C91—H91C	109.5
S2—C13—S1	119.95 (7)	H91B—C91—H91C	109.5
C15—C14—S1	113.08 (9)	O93—C92—H92A	109.9
O3—C15—O2	124.07 (12)	C91—C92—H92A	109.9
O3—C15—C14	125.39 (12)	O93—C92—H92B	109.9
O2—C15—C14	110.52 (10)	C91—C92—H92B	109.9
C15—O2—C16'	122.7 (4)	H92A—C92—H92B	108.3
C15—O2—C16	112.09 (14)	O93—C94—H94A	109.9
C16'—O2—C16	19.7 (3)	C95—C94—H94A	109.9
C17—C16—O2	109.5 (2)	O93—C94—H94B	109.9
O2—C16'—C17'	98.6 (5)	C95—C94—H94B	109.9
N2—C18—C12	179.36 (14)	H94A—C94—H94B	108.3
O93—C92—C91	108.82 (12)	C94—C95—H95A	109.5
C92—O93—C94	112.91 (11)	C94—C95—H95B	109.5
O93—C94—C95	109.05 (12)	H95A—C95—H95B	109.5
C11—N1—H01	116.6 (10)	C94—C95—H95C	109.5
C1—N1—H01	116.8 (10)	H95A—C95—H95C	109.5
C1—C2—H2	119.8	H95B—C95—H95C	109.5
C3—C2—H2	119.8	Ca—O1W—H1W	138.0 (12)
C4—C3—H3	119.7	Ca—O1W—H2W	113.9 (13)
C2—C3—H3	119.7	H1W—O1W—H2W	105.2 (16)
C3—C4—H4	119.6	Ca—O2W—H3W	129.2 (12)
C9—C4—H4	119.6	Ca—O2W—H4W	122.9 (13)
C6—C5—H5	119.2	H3W—O2W—H4W	107.1 (16)
			
O1^i^—Ca—O1—C11	71 (6)	Ca—O1—C11—C12	79.9 (3)
O2W^i^—Ca—O1—C11	153.0 (3)	C1—N1—C11—O1	3.5 (2)
O2W—Ca—O1—C11	−27.0 (3)	C1—N1—C11—C12	−176.62 (11)
O1W^i^—Ca—O1—C11	−119.3 (3)	O1—C11—C12—C13	−176.38 (12)
O1W—Ca—O1—C11	60.7 (3)	N1—C11—C12—C13	3.71 (19)
C11—N1—C1—C2	−34.63 (18)	O1—C11—C12—C18	3.10 (16)
C11—N1—C1—C10	145.94 (12)	N1—C11—C12—C18	−176.80 (11)
N1—C1—C2—C3	179.07 (11)	C18—C12—C13—S2	−177.98 (9)
C10—C1—C2—C3	−1.52 (19)	C11—C12—C13—S2	1.48 (19)
C1—C2—C3—C4	0.79 (19)	C18—C12—C13—S1	1.28 (15)
C2—C3—C4—C9	0.62 (19)	C11—C12—C13—S1	−179.27 (10)
C9—C5—C6—C7	−0.3 (2)	C14—S1—C13—C12	178.99 (9)
C5—C6—C7—C8	0.4 (2)	C14—S1—C13—S2	−1.70 (9)
C6—C7—C8—C10	0.1 (2)	C13—S1—C14—C15	−68.69 (9)
C3—C4—C9—C5	178.77 (13)	S1—C14—C15—O3	−20.07 (15)
C3—C4—C9—C10	−1.27 (19)	S1—C14—C15—O2	161.69 (8)
C6—C5—C9—C4	179.68 (13)	O3—C15—O2—C16'	−11.5 (4)
C6—C5—C9—C10	−0.29 (19)	C14—C15—O2—C16'	166.8 (3)
C7—C8—C10—C9	−0.74 (19)	O3—C15—O2—C16	7.3 (2)
C7—C8—C10—C1	179.59 (12)	C14—C15—O2—C16	−174.45 (16)
C4—C9—C10—C8	−179.15 (12)	C15—O2—C16—C17	170.4 (2)
C5—C9—C10—C8	0.82 (17)	C16'—O2—C16—C17	−63.0 (12)
C4—C9—C10—C1	0.53 (17)	C15—O2—C16'—C17'	103.4 (5)
C5—C9—C10—C1	−179.50 (12)	C16—O2—C16'—C17'	41.3 (9)
C2—C1—C10—C8	−179.49 (12)	C13—C12—C18—N2	162 (14)
N1—C1—C10—C8	−0.05 (18)	C11—C12—C18—N2	−17 (14)
C2—C1—C10—C9	0.84 (17)	C91—C92—O93—C94	179.86 (11)
N1—C1—C10—C9	−179.72 (10)	C92—O93—C94—C95	−179.38 (12)
Ca—O1—C11—N1	−100.2 (3)		

Symmetry codes: (i) −*x*, −*y*+1, −*z*+2.

### Hydrogen-bond geometry (Å, °)

**Table:**

*D*—H···*A*	*D*—H	H···*A*	*D*···*A*	*D*—H···*A*
N1—H01···S2	0.855 (16)	2.279 (16)	3.0274 (11)	146.2 (14)
O1W—H1W···O93	0.82 (1)	1.94 (1)	2.7531 (14)	171 (2)
O1W—H2W···N2^i^	0.82 (1)	2.22 (1)	2.9902 (15)	157 (2)
O2W—H3W···O3^ii^	0.82 (1)	2.03 (1)	2.7797 (13)	151 (2)
O2W—H4W···S2^ii^	0.81 (1)	2.51 (1)	3.2666 (11)	157 (2)

Symmetry codes: (i) −*x*, −*y*+1, −*z*+2; (ii) *x*−1, *y*, *z*.
